# A New Approach for Accurate Detection of Chromosome Rearrangements That Affect Fertility in Cattle

**DOI:** 10.3390/ani10010114

**Published:** 2020-01-10

**Authors:** Rebecca L. Jennings, Darren K. Griffin, Rebecca E. O’Connor

**Affiliations:** School of Biosciences, University of Kent, Giles Lane, Canterbury CT2 7NJ, UK; rj267@kent.ac.uk (R.L.J.); d.k.griffin@kent.ac.uk (D.K.G.)

**Keywords:** cattle, translocation, FISH, artificial insemination, subfertility, chromosome, genetics

## Abstract

**Simple Summary:**

Globally, cattle production has more than doubled since the 1960s, with widespread use of artificial insemination (AI) and an emphasis on a small pool of high-genetic-merit animals. Selecting AI bulls with optimal fertility is therefore vital, as impaired fertility reduces genetic gains and reduces production, resulting in heavy financial and environmental losses. Chromosome translocations, where large parts of the genome are inappropriately attached in abnormal patterns, are a common cause of reduced fertility; however, reciprocal translocations are significantly underreported due to the difficulties inherent in analysing cattle chromosomes. Based on our previous work, we have developed an approach for the unambiguous detection of abnormalities that affect fertility. We applied this method on the chromosomes of 39 bulls, detecting multiple abnormalities that affect fertility, including those that would be undetectable using traditional screening techniques. With UK dairy calving rates of only 50–60%, it is vital to reduce further fertility loss in order to maximise productivity. The approach developed here identifies abnormalities that DNA sequencing will not, and has the potential to lead to long-term gains, delivering meat and milk products in a more cost-effective and environmentally-responsible manner to a growing population.

**Abstract:**

Globally, cattle production has more than doubled since the 1960s, with widespread use of artificial insemination (AI) and an emphasis on a small pool of high genetic merit animals. Selecting AI bulls with optimal fertility is, therefore, vital, as impaired fertility reduces genetic gains and production, resulting in heavy financial and environmental losses. Chromosome translocations, particularly the 1;29 Robertsonian translocation, are a common cause of reduced fertility; however, reciprocal translocations are significantly underreported due to the difficulties inherent in analysing cattle chromosomes. Based on our porcine work, we have developed an approach for the unambiguous detection of Robertsonian and reciprocal translocations, using a multiple-hybridization probe detection strategy. We applied this method on the chromosomes of 39 bulls, detecting heterozygous and homozygous 1;29 translocations and a 12;23 reciprocal translocation in a total of seven animals. Previously, karyotype analysis was the only method of diagnosing chromosomal rearrangements in cattle, and was time-consuming and error-prone. With calving rates of only 50–60%, it is vital to reduce further fertility loss in order to maximise productivity. The approach developed here identifies abnormalities that DNA sequencing will not, and has the potential to lead to long-term gains, delivering meat and milk products in a more cost-effective and environmentally-responsible manner to a growing population.

## 1. Introduction

On a global scale, cattle meat and milk production has more than doubled between 1961 and 2014, increasing from 28 million to 68 million tonnes per year for meat products, and 344 million to 792 million tonnes for milk products [1]. To support this increasing demand, the use of artificial insemination (AI) has become widespread in the cattle breeding industry. In many breeding programmes, the emphasis on genomic selection is on genotype and pedigree analysis, with relatively little attention being paid to the underlying fertility of the animal. However, the extensive use of artificial insemination using a small pool of high genetic-merit bulls, and the rising use of in vitro-produced embryos, means that the importance of selecting parents that also have optimal fertility is vital. Impaired fertility reduces genetic gain, increases veterinary costs, and reduces milk and meat production, all of which result in heavy financial and environmental losses for the breeding company; costs that is ultimately passed on to the end consumer.

Semen analysis is commonly used as a fertility indicator in livestock breeding programmes where volume, morphology, motility and concentration are routinely measured [2]. However, this type of analysis is thought to be an unreliable indicator of fertility, and does not allow for the detection of underlying subfertility on a chromosomal level [3]. Instead, the most widely used parameter for the detection of subfertility in cattle is the ‘nonreturn rate’ (the number of females returning to the oestrus cycle, being therefore indicative of a failure to conceive) [4].

Chromosomal rearrangements, including Robertsonian (centromeric end fusion) and reciprocal (nonhomologous exchange) translocations, can have a significant detrimental effect on the fertility of cattle. Where these rearrangements occur, the process of meiotic pairing and chromosome segregation during gametogenesis is disturbed, leading to gametes that can be genetically unbalanced [5]. These unbalanced gametes inevitably result in early embryonic loss due to reduced viability. In recent decades, efforts have been made to diagnose fertility issues in domestic breeding animals using chromosome analysis. In 1964, Ingemar Gustavsson first reported the presence of the 1;29 centromeric fusion (Robertsonian translocation) in a population of Swedish Red and White cattle [6]. Since then, the 1;29 translocation has been the most commonly seen rearrangement of the 44 that have been identified in cattle so far [7], with cases found in all breeds, except Holstein-Fresian [8]. In one 15-year study of the Italian breeding population, 7.1% animals were identified as carrying a Roberstonian translocation [9]. These heterozygous 1;29 carriers are phenotypically normal, but suffer a reduction in fertility of 3–5% [10]. Homozygous carriers are rare, but have been reported by several groups, although incidence varies between breeds. Reported examples of the homozygous 1;29 state include the presence in 8.5% of Blonde d’Aquitaine bulls [10], along with cases found in five of the eight Portuguese cattle breeds [11]. All cattle (*Bos taurus* and *Bos indicus*) have the same chromosome complement with 2n = 60, and thus, any novel approach must be applicable to all commercial breeds.

Reciprocal translocations have been identified in cattle, albeit much less frequently, with the aforementioned Italian study reporting a rate of 0.03% [9]. To date, only 19 reciprocal translocations involving different chromosomes in cattle have been reported [12]. In fact, De Lorenzi and colleagues suggested that the frequency of reciprocal translocations is grossly underreported, largely due to the inherent difficulties in detecting these rearrangements using routine cytogenetics [12]. The cattle karyotype is notoriously difficult to analyse reliably because of a diploid number of 60, largely made up of similar-sized acrocentric chromosomes, and is therefore problematic for the detection of anything other than Robertsonian translocations. A molecular approach that will detect reciprocal and Robertsonian translocations is, therefore, essential.

Recently, we developed an approach for the detection of cryptic and overt translocations in boars [13]. This method uses a panel of subtelomeric fluorescence *in-situ* hybridisation (FISH) probes on a multihybridisation device as a means of highlighting the ends of each chromosome, thereby facilitating the identification of rearrangements between chromosomes. The purpose of this study was to use similar technology to isolate and visualise each end of every chromosome in cattle. Here, as proof of principle, we use a small sample size; however, all cattle have the same chromosomes, thereby allowing for the detection of translocations, particularly the challenging reciprocal ones.

## 2. Materials and Methods

Heparinised whole blood samples from 39 Holstein bulls were obtained from local suppliers. Samples were collected as part of standard procedures used for commercial evaluation by in house trained veterinarians via standard phlebotomy in heparin tubes. Whole blood samples were cultured for 72 h in PB MAX Karyotyping medium (Gibco, Thermo Fisher Scientific, Waltham, MA, USA) at 37 °C, 5% CO_2_. Cell division was arrested by the addition of colcemid at a concentration of 10.0 μg/mL (Gibco, Thermo Fisher Scientific, Waltham, MA, USA) for 35 min prior to hypotonic treatment with 75 M KCl and fixation to glass slides using 3:1 methanol:acetic acid.

### 2.1. Selection and Preparation of Fluorescence In-Situ Hybridisation (FISH) Probes

Bacterial artificial chromosome (BAC) clones of approximately 150 kb in size were selected from the Btau 4.6.1 NCBI database (www.ncbi.nlm.nih.gov) and ordered from the CHORI-240 Bovine BAC library for each autosome and the X chromosome (see Table 1). A lack of available BACs for the Y chromosome meant that this chromosome was excluded from the study. BAC DNA was isolated using the Qiagen Miniprep Kit (Qiagen, Hilden, Germany), the products of which were then amplified and directly labelled by nick translation with FITC-Fluroescein-12-UTP (Roche, Basel, Switzerland) for subcentromeric probes and Texas Red-12-dUTP (Invitrogen, Thermo Fisher Scientific, Waltham, MA, USA) for distal q-arm probes prior to purification. A list of BACs is given in Table 1.

### 2.2. Development of Multiprobe Device

**Figure 1:**
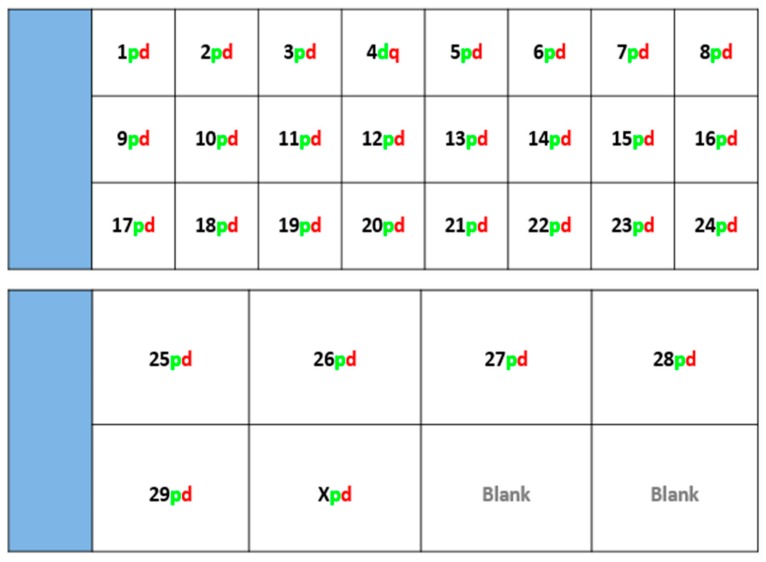
Schematic demonstrating the layout of probes designed to map to each bovine chromosome-selected from the most proximal (p) and most distal region (d) of each individual chromosome.

Fluorescently-labelled probes were diluted to a concentration of 10 ng/μL in sterile distilled water along with competitor DNA (Bovine Hybloc, Applied Genetics Laboratories, Melbourne, FL, USA). Each probe combination contained a probe isolated from each end of the chromosome, and was individually assigned with the appropriate chromosome number followed by the letter p (proximal) or d (distal). Using a proprietary Chromoprobe Multiprobe System device manufactured by Cytocell Ltd., Cambridge, UK, each probe combination (e.g., 1pd) for chromosomes 1 to 24 was air dried onto a square of the device. The corresponding glass slide was subdivided into 24 squares, designed to align to the 24 squares on the device upon which chromosome suspensions were fixed. A second 8-square device was used to facilitate the larger number of chromosomes in the cattle karyotype. The precise orientation of the clones and development of the bespoke device is given in the results section (Figure 1).

### 2.3. Fluorescence In-Situ Hybridisation (FISH)

Slides were dropped with fixed metaphase preparations and dehydrated through an ethanol series (2 min each in 2× sodium saline citrate (SSC), 70%, 85% and 100% ethanol at room temperature). Formamide-based hybridisation buffer (Cytocell Hyb I, Cambridge, UK) was pipetted onto each square of the device in order to resuspend the probes. The glass slide and the device were sandwiched together and warmed on a 37 °C hotplate for 10 min. The probe and target DNA were subsequently denatured on a 75 °C hotplate for 5 min prior to overnight hybridisation in a dry hybridisation chamber in a 37 °C water bath. Slides were washed post hybridisation for 2 min in 0.4× SSC at 72 °C and 30 s in 2× SSC/0.05% Tween 20 at room temperature, then counterstained using DAPI in VECTASHIELD. Metaphases for karyotyping were stained with DAPI in VECTASHIELD antifade medium (Vector Laboratories, Peterborough, UK). Image capturing was performed using an Olympus BX61 epifluorescence microscope (Olympus, Tokyo, Japan) with a cooled CCD camera and SmartCapture (Digital Scientific UK, Cambridge, UK) system. The SmartType software (Digital Scientific UK, London, UK) was used for karyotyping purposes.

## 3. Results

### 3.1. Generation and Validation of a Device and Scheme Capable of Detecting Reciprocal and Robertsonian Translocations in Cattle

Using technology adapted for translocation screening in pigs, the screening device was arranged as shown in Figure 1, where, for each autosome, a proximal (near the centromere) and distal probe were labelled in green and red respectively to highlight the ends of each chromosome. For the X chromosome, the proximal probe was located in a subtelomeric position at the end of the p arm and one at the distal end of the q-arm. Given the number of cattle chromosomes (2n = 60), a 24-square (as per the porcine device) plus an extra 8-square device was used.

### 3.2. Validation of Device and Karyotypes

**Figure 2:**
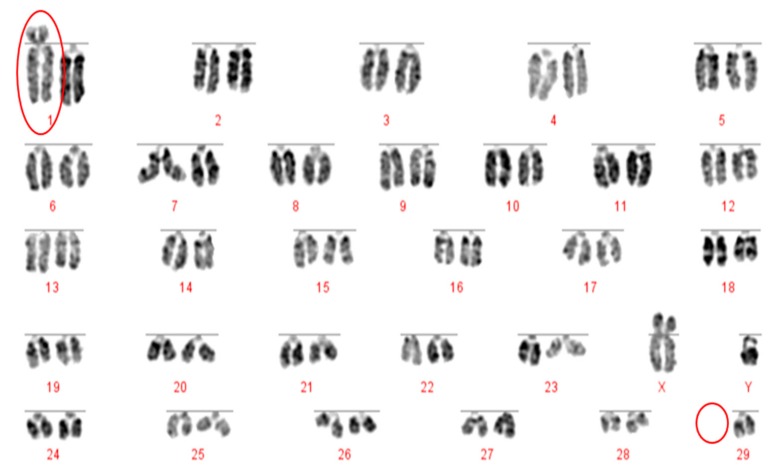
DAPI stained karyotype of a 2n = 59 bull with a rob (1;29). Robertsonian translocation and the missing chromosome 29 are circled in red.

**Figure 3:**
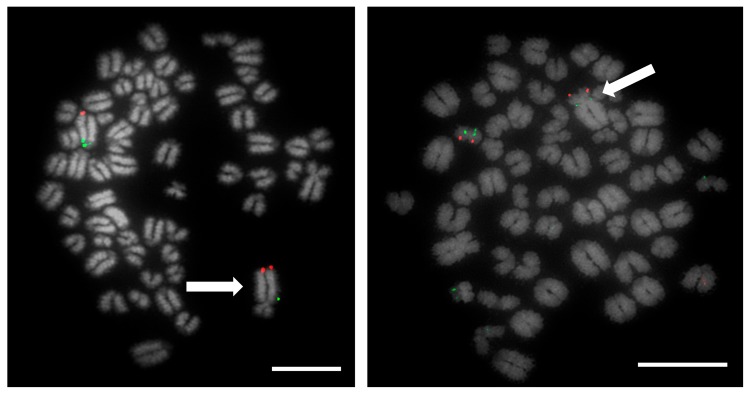
Metaphase spread of heterozygous 1;29 translocation carrier. Left image shows labelled FISH probes for chromosome 1, where CH240-321O2 (FITC) is the proximal probe and CH240-96M6 (TxRed) is the distal probe. The translocation is marked by an arrow. Right image shows labelled FISH probes for chromosome 29, where CH240-367D17 (FITC) represents the proximal probe and CH240-257F23 (TxRed) maps to the distal end- The translocation is marked by an arrow. Scale bar 10 µm.

**Figure 4:**
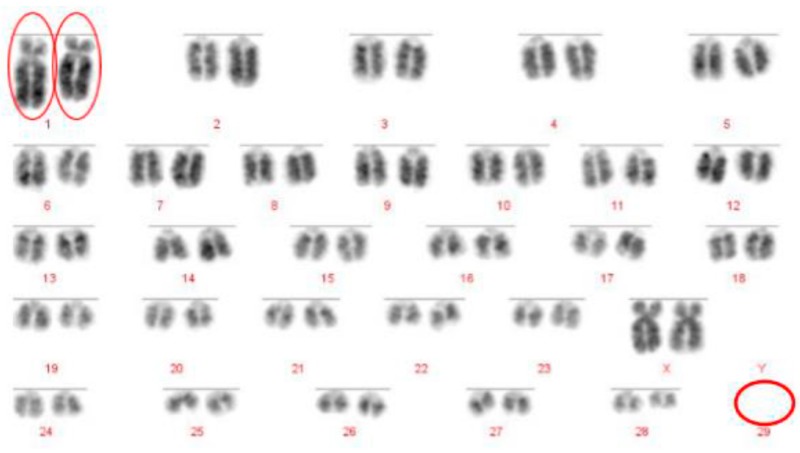
DAPI stained metaphase chromosomes of a homozygous 1;29 Robertsonian translocation in a British White bull (2n = 58, XX). Homozygous Robertsonian translocation (1;29) circled in red. Diagnosis confirmed by FISH.

**Figure 5:**
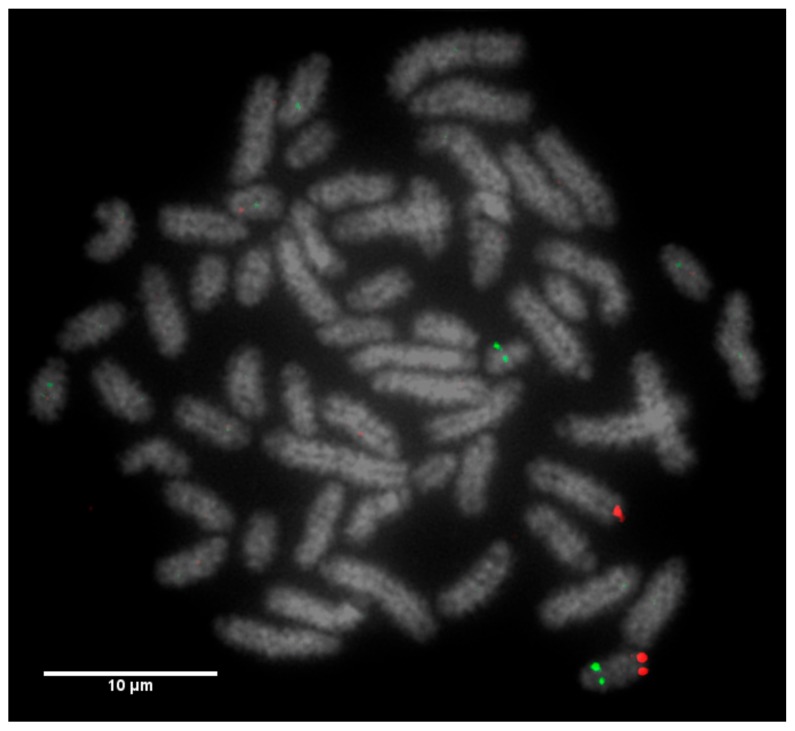
Labelled FISH probes for chromosome 23, where the proximal probe is CH240-102P19 (FITC) and the distal probe is (CH240-374G6 (Texas Red). A misplacement of signals illustrates a reciprocal translocation between chromosome 12 and chromosome 23.

A total of 39 bulls were screened using both karyotyping and the FISH multiprobe method, the results of which are show in Table 2. Bright signals were seen in each of the hybridization squares, with five animals revealing a 1;29 translocation. Three of these were heterozygous, and two were homozygous. In two samples, the FISH method revealed a reciprocal translocation (rcp 12;23), thereby demonstrating that karyotypically-undetectable reciprocal translocations can be identified using this technology. The results are given in Figure 2, Figure 3, Figure 4 and Figure 5 and summarized in Table 2.

No reciprocal translocations were identified by karyotype analysis, although two carriers were identified using FISH. The translocation identified involved chromosomes 12 and 23, with the same translocation affecting two bulls, as shown in Figure 5.

## 4. Discussion

The consequences of using a subfertile bull in an AI breeding programme are many. First of all, while using a subfertile bull may result in a small number of pregnancies, the pregnancy rates will inevitably be significantly lower than expected. This then leads to extended calving intervals and an increased likelihood that a higher proportion of cows will be culled for presumed sterility. Both of these factors result not just in reduced financial returns, but also in a large degree of wastage, raising ethical and environmental concerns. This is particularly important when the very large bull to cow ratios employed in AI programmes are taken into account. With average calving rates of only 50–60% in UK domestic dairy cattle, it is vital to prevent any further potential loss of fertility in order to maximise the opportunities for each cow to conceive and to improve productivity [14].

Prior to the results reported here, standard karyotype analysis, a time-consuming and error prone method, was the only means of diagnosing chromosomal rearrangements in cattle. De Lorenzi and colleagues calculated that for a translocation to be observable through karyotyping alone, an abnormal chromosome derivative must be either at least 15% (185 Mb) longer than chromosome 1, or 40% (26.4 Mb) shorter than chromosome 25 [12]. Even with optimum G-banding preparations, it is likely that most reciprocal translocations involving chromosomes 2–24 would be indistinguishable from other autosomes. It is vital, therefore, that efficient and accurate methods are implemented for the detection of chromosome translocations as part of a routine screening programme for cattle destined for AI. The results generated in this study demonstrate the validity of a FISH-based screening device for the detection of reciprocal translocations, two of which would remain undiagnosed if standard karyotyping alone had been used. Efforts to eradicate chromosomal translocations from the cattle breeding herd are ongoing; however, the results presented here demonstrate that both Robertsonian and reciprocal translocations are present in the breeding population. Many of these may be de novo rearrangements, but it is highly likely that the reciprocal translocation identified here is one that has been carried through multiple generations but which had not been identified due to the inherent difficulties in screening using traditional methods. Screening for chromosomal translocations that result in economic loss is, therefore, more important than ever, and this study demonstrates, in only a small group of animals, a means by which it could be achieved in the future.

The Robertsonian translocations identified in this study, while detectable with basic karyotyping, can also easily and accurately be identified using FISH. Interestingly, despite great efforts in many breeding programmes to eliminate the 1;29 translocation, our results suggest that either these efforts have not been wholly successful, or that this rearrangement continues to recur de novo. Without historical data and ongoing routine screening of all animals entering the breeding population, it is difficult to ascertain what proportion of these rearrangements fall into this category.

This paper provides a small proof of principle for an approach that could potentially have wide applicability. The development and implementation of this FISH-based assay has, however, already markedly improved the efficiency and accuracy of translocation screening, allowing multiple hybridisation experiments in a single assay. Whilst chromosomal translocations have been demonstrated to significantly affect fertility in all species tested, in cattle, many of these rearrangements have remained undetected due to the inherent difficulties in finding them using previous technologies. The method presented here resolves this issue, allowing for the rapid identification of an abnormality, and corresponding rapid removal of affected animals from the herd. Not only will this lead to a reduction in the economic losses associated with using a subfertile bull, it will also reduce the need for the unnecessary culling of cows and bulls that are suspected (but unproven) to be sterile, thereby reducing economic and environmental loss. Moreover, the karyotype of both *Bos Taurus* and *Bos indicus* is identical (aside from the morphology of the Y chromosome), and therefore, although only established on a small number of individuals, this approach is universally applicable to all commercial breeding bulls.

In addition, an increasing emphasis on the use of in vitro production (IVP) methods to improve cattle breeding means that the requirement for high genetic merit gametes is not just limited to the analysis of bulls, but that the need to screen cows, and ultimately oocytes or embryos, for chromosome abnormalities will also become increasingly important [15]. This screening approach allows both donor parents to be screened for underlying chromosomal abnormalities prior to their use in IVP programmes, thereby improving the genetic quality of the embryos generated using these methods. Other efforts in our laboratory allow for the screening of oocytes and embryos [15].

Finally, with the success of this screening method in place and the success of our previously developed method in screening for chromosome abnormalities in pigs, it is plausible to suggest that this technique could be applied to any animal of interest, with the horse being an ideal future candidate. The domestic horse (2n = 64) is of significant interest to many different groups worldwide; the thoroughbred breeding industry, for example, could benefit from a similar screening service. Previous cytogenetic studies have identified chromosomal translocations that affect fertility in thoroughbred mares [16], another group for which breeders place a significant importance on high genetic merit in the breeding population. Having the tools to examine and diagnose chromosomal abnormalities in a similarly fast and efficient manner would, therefore, be beneficial to this industry.

## 5. Conclusions

The approach developed here has the potential to lead to long-term improved productivity, delivering meat and milk products in a more cost-effective and environmentally-responsible manner to a growing population. The widespread use of artificial insemination and IVP, and the large market for superior bull semen being sold to both small and large-scale cattle breeding operations suggests that improvements in productivity will have a wide impact. This will affect not only large commercial breeders, but also smaller farmers, for whom reduced wastage per animal may be more critical.

## Figures and Tables

**Table 1:** Cattle BACs by chromosome from the CHORI-240 library.

Chrom	Arm	Clone Name	Span	Chrom	Arm	Clone Name	Span (bp)
1	p	CH240-321O2	179,965	16	p	CH240-139M7	166,377
	d	CH240-96M6	187,920		d	CH240-315I10	186,228
2	p	CH240-457J20	198,157	17	p	CH240-267P22	176,654
	d	CH240-227E16	179,789		d	CH240-313I20	182,729
3	p	CH240-154A5	174,225	18	p	CH240-14C14	163,878
	d	CH240-302G6	190,291		d	CH240-436N22	179,260
4	p	CH240-416O20	170,609	19	p	CH240-349G17	169,018
	d	CH240-193F3	179,112		d	CH240-390C5	180,283
5	p	CH240-326L8	188,525	20	p	CH240-394L14	182,595
	d	CH240-248M21	163,993		d	CH240-339K22	183,557
6	p	CH240-324B6	180,970	21	p	CH240-301D14	163,699
	d	CH240-5F18	184,848		d	CH240-62O23	176,169
7	p	CH240-415D2	182,547	22	p	CH240-426O23	182,818
	d	CH240-276L16	168,781		d	CH240-313B20	173,299
8	p	CH240-443K7	175,465	23	p	CH240-102P19	179,615
	d	CH240-241A18	176,318		d	CH240-374G6	174,942
9	p	CH240-25A3	177,086	24	p	CH240-382F1	171,530
	d	CH240-298I24	172,331		d	CH240-19L13	171,917
10	p	CH240-421B11	166,378	25	p	CH240-198J4	186,545
	d	CH240-325F16	179,292		d	CH240-379D22	163,818
11	p	CH240-314K5	165,445	26	p	CH240-428I10	181,997
	d	CH240-344O3	183,795		d	CH240-389H1	176,691
12	p	CH240-261C16	164,440	27	p	CH240-7G11	184,155
	d	CH240-262C4	165,223		d	CH240-352M8	184,694
13	p	CH240-461F6	188,788	28	p	CH240-313L4	181,707
	d	CH240-471M8	178,736		d	CH240-63D12	183,932
14	p	CH240-319C15	181,738	29	p	CH240-367D17	179,713
	d	CH240-240M1	178,587		d	CH240-257F23	188,054
15	p	CH240-225A24	151,902	X	p	CH240-121E1	176,736
	d	CH240-386C2	168,728		q	CH240-472J20	186,872

**Table 2:** Summary of results from screening 39 animals using karyotyping and FISH.

Diagnosis	Numbers	Method of Detection
Heterozygous Robertsonian (1;29)	3	Karyotype-confirmed with FISH
Homozygous Robertsonian (1;29)	2	Karyotype-confirmed with FISH
Reciprocal (12;23)	2	FISH-karyotype appeared normal
Normal	32	Karyotype, FISH
